# Social Vulnerability and Medical Complexity Among Medicare Beneficiaries Receiving Home Health Without Prior Hospitalization

**DOI:** 10.1093/geroni/igaa049

**Published:** 2020-10-03

**Authors:** Julia G Burgdorf, Tracy M Mroz, Jennifer L Wolff

**Affiliations:** 1 Department of Health Policy and Management, Johns Hopkins Bloomberg School of Public Health, Baltimore, Maryland; 2 Department of Rehabilitation Medicine, University of Washington School of Medicine, Seattle

**Keywords:** Home care, Home care services, Medicare, Older adult

## Abstract

**Background and Objectives:**

Recent Medicare home health payment changes reduce reimbursement for care provided to patients without a preceding hospitalization. Beneficiaries may enter home health without a preceding hospitalization via referral from a community provider or through incurring multiple episodes of home health care. We assess potential implications of this change by examining the characteristics of patients accessing Medicare home health through each of these pathways.

**Research Design and Methods:**

Nationally representative retrospective cohort study of 1,224 (weighted *n* = 5,913,080) older adults who participated in the National Health and Aging Trends Study between 2011 and 2015 and received Medicare-funded home health within 1 year of interview. Patient characteristics before home health were drawn from the National Health and Aging Trends Study, while characteristics during home health, referral source, and number of episodes incurred were drawn from linked Outcomes and Assessment Information Set and Medicare claims. We tested for differences in characteristics by referral source and number of episodes using weighted chi-square tests and *t* tests.

**Results:**

Patients referred to home health from the community were more than twice as likely to be Medicaid-enrolled (24.0% vs 12.5%, *p* < .001), have dementia (29.5% vs 12.4%, *p* < .001), and have received 80 or more hours/month of family caregiver assistance (20.7% vs 10.1%, *p* < .001) prior to home health entry compared to those referred from a hospital or skilled nursing facility. Patients who incurred multiple episodes in a spell of home health care were more likely to have high clinical severity during home health (48.3% vs 28.1%, *p* < .001), compared to those with a single episode.

**Discussion and Implications:**

Greater social vulnerability and care needs *before* home health were associated with community referral, while greater clinical severity *during* home health was associated with incurring multiple episodes of care. Findings suggest that recent payment changes may threaten home health access among beneficiaries with greater social vulnerability and/or medical complexity.

**Translational Significance:** Medicare beneficiaries receiving home health care without a preceding hospitalization have significant social vulnerability and clinical severity. Reducing reimbursement for home health care provided without a preceding hospitalization may threaten vulnerable beneficiaries’ ability to meet their care needs. The Centers for Medicare and Medicaid Services should carefully monitor whether recent reimbursement changes disproportionately reduce access to care for select groups of high-need beneficiaries.

## Background and Objectives

The Medicare-funded home health benefit offers home-based care for homebound beneficiaries requiring intermittent skilled nursing or therapy (1). This care is delivered and paid for episodically; episodes begin with the first home health visit and continue for 30 days (until 2020, episodes were 60 days), at which point a patient must be recertified to continue receiving care (2). Traditionally structured as a postacute benefit (3), utilization has changed in recent years such that a growing proportion of home health episodes occur without an immediately preceding hospitalization (4). This development is the result of two coinciding trends: an increase in referrals to home health from the community and an increase in those receiving multiple successive episodes of home health care. These trends have drawn attention amid discussions of how broad eligibility criteria may contribute to ineffective use of the home health benefit (5), ongoing concerns regarding high margins and fraudulent practices of some home health providers (1,6), and striking growth in Medicare spending on home health, which more than doubled between 2000 and 2016 (1).

In January 2020, the Centers for Medicare and Medicaid Services (CMS) implemented a new prospective payment system for home health, the Patient-Driven Groupings Model (PDGM), which lowers reimbursement for episodes not immediately preceded by a hospitalization. CMS estimates that, under PDGM, average reimbursement for these episodes will decrease by 11%, holding all other patient characteristics constant (7). Two types of patients will be affected by this payment change: (a) those referred to home health by a community provider such as a primary care physician and (b) those who incur multiple successive episodes, regardless of initial referral source (7,8).

It is important to understand the characteristics of patients who may be affected by PDGM changes which reduce reimbursement for episodes not immediately preceded by hospitalization. Historically, home health providers have been highly responsive to payment system revisions (3,9–11). Previous changes in reimbursement have disproportionately reduced access for beneficiaries with greater disability, frailty, and social vulnerability (9,11,12), defined as the gap between an individual’s available resources and their life challenges (13). Factors associated with greater social vulnerability include lower socioeconomic status, nonwhite race, and cognitive impairment. Three prior studies have examined differences in patient characteristics by home health referral source. Mroz et al. (14) found that community-admitted patients were significantly more likely to be nonwhite, Medicaid-enrolled, and have cognitive impairment; however, the sample was restricted to residents of rural areas. Fout et al. (15) found that a greater proportion of community-admitted patients had cognitive impairment and were Medicaid-enrolled, and Wysocki and Cheh (6) found that community-admitted patients were significantly more likely to be Medicaid-enrolled and live in a state with high levels of home health provider fraud/abuse. However, none of these studies examined contextual factors measured before the home health episode or examined differences in patient characteristics by both referral source and number of episodes.

Our study fills two remaining gaps in the available literature regarding variation in home health patient characteristics between those with versus without an immediately preceding hospitalization. First, although contextual factors, such as availability of family caregivers, may affect home health care utilization and represent another facet of social vulnerability, all previous work has been restricted to measures gathered *during* the home health episode, missing potentially valuable information about patients’ social contexts (6,14,15). We draw on a unique analytic data set that links a nationally representative survey with comprehensive information on individual health, functional disability, and caregiver support before home health, to patient assessments conducted by home health clinicians during the episode of care and Medicare claims. By describing differences in contextual factors present before the home health episode, we are able to more fully characterize the population likely to be affected by recent PDGM reimbursement changes.

Second, the recent reduction in reimbursement affects two groups of patients, as described above: those initially referred from the community and those receiving multiple successive episodes. Prior research found that those initially admitted from the community were also more likely to receive multiple episodes of care (6,15). However, no previous work has separately examined these two pathways into home health to untangle whether observed variation in patient characteristics is driven by differences based on referral source, number of episodes, or both. To better understand the type of patient likely to be affected by this reimbursement reduction, we examine whether the same underlying characteristics are associated with community referral and receiving multiple episodes. Our study aims to describe the characteristics of Medicare home health patients, comparing patients by referral source and number of episodes received. Findings provide new information regarding variation in home health patient characteristics, informing our understanding of which Medicare beneficiaries are most likely to be affected by the recent implementation of PDGM.

## Research Design and Methods

### Data Sources

Data were drawn from the National Health and Aging Trends Study (NHATS) and linked Outcomes and Assessment Information Set (OASIS) Version C, and Medicare claims. NHATS is an annual, nationally representative, in-person survey of Medicare beneficiaries aged 65 and older. NHATS comprehensively assesses older adults’ sociodemographic characteristics, health and functional status, and social supports (16). OASIS is a mandatory, standard patient assessment completed by home health clinicians and reported to CMS during a Medicare home health episode. OASIS assessments contain information regarding the patient’s clinical and functional status, plan of care, and family caregiver support while receiving home health. OASIS assessments are used by providers to inform care planning and by CMS to determine home health reimbursement and support public reporting of home health provider quality (17).

### Sample

Our analytic sample included community-dwelling older adults who participated in NHATS between 2011 and 2015 and received home health care within 1 year of the interview. We excluded NHATS participants who did not use home health services during this period, as well as those living in congregate settings such as assisted living, due to the availability of supports that may affect home health utilization. As the PDGM is specific to fee-for-service Medicare, our sample excludes Medicare Advantage enrollees. Of the 8,245 NHATS respondents included in the initial 2011 survey wave, 1,758 accessed Medicare home health within 12 months of the initial (2011) or a follow-up (2012–2015) survey interview and were community-dwelling and enrolled in fee-for-service Medicare in the year they received home health.

We linked 2011–2015 NHATS surveys with 2011–2016 OASIS assessments and Medicare home health claims. For each participant, the Start of Care OASIS assessment from the index home health episode was identified and linked to the NHATS survey immediately preceding the index home health episode. As we exclusively examine the index episode, each participant appears in the sample only once. The average gap in time between the NHATS survey and the OASIS assessment was 6.5 months (*SD* = 3.3), giving us access to information about contextual factors present before the home health episode, as well as immediate factors during the home health episode, that may affect utilization patterns.

### Measures

Our key independent variables represent the two possible pathways into receiving home health without a preceding hospitalization: initial referral from the community or receiving multiple successive episodes of home health care (8). Patients were considered to have a postacute referral if they received inpatient care in an acute care hospital or skilled nursing facility in the 14 days prior to their index home health episode and a community referral otherwise. Patients were categorized as having incurred a single episode of care if they received no additional home health episodes within 60 days of the end of the index episode and as having incurred multiple episodes otherwise. As PDGM reduces reimbursement for all later episodes in a spell of care, regardless of how many are incurred, we do not control for the actual number of episodes received.

Older adults’ sociodemographic characteristics, health status, and caregiver availability and assistance *prior to home health* were drawn from NHATS. Sociodemographic characteristics included self-reported age, sex, race, and Medicaid enrollment. Measures of health status included self-reported health, numbers of chronic medical conditions, and hospitalization in the prior year, as well as a measure of probable dementia determined from self-reported physician diagnosis of Alzheimer’s or dementia, proxy respondent responses to a dementia screening tool, and older adult performance on cognitive tests in the NHATS, as described previously (18). Caregiver availability and assistance included the number of family caregivers, hours per month of assistance received from family caregivers, and types of help received from family caregivers, including assistance with household tasks, mobility, self-care, and medication management.

Information about patients’ cognitive and functional impairment and health status *during home health* was drawn from the OASIS. Cognitive impairment was measured by the home health clinician’s characterization of the patient’s current level of alertness, orientation, comprehension, concentration, and immediate memory for simple commands (OASIS item M1700 (17)). Measures of functional impairment and clinical severity are drawn from Health Insurance Prospective Payment System codes used by Medicare to adjust home health payments based on patient characteristics. These measures are based on extensive research and testing sponsored by CMS and are derived from multiple OASIS items to give a holistic view of patient status and care needs (19,20).

### Analysis

We described study participants’ sociodemographic characteristics, health status, and caregiver availability and assistance prior to home health, and health and functional status during home health, comparing those with a preceding hospitalization to those without. We used weighted Satterthwaite Rao–Scott chi-square tests of independence to test for differences between groups on categorical variables and weighted *t* tests to test for differences between groups on continuous variables. All analyses employed survey weights and design variables provided by NHATS to account for complex survey design and were performed in SAS 9.4 (SAS Institute, Inc., Cary, NC.).

We sought to isolate the variation in characteristics attributable to each of the two pathways into home health without a preceding hospitalization: community versus postacute referral and incurring multiple episodes versus a single episode. To do this, we first compared participant characteristics based on referral source, while stratifying by the number of home health episodes incurred. This approach allowed us to present differences associated with community referral source only. We then compared participant characteristics based on whether the individual incurred multiple episodes or a single episode, while stratifying by referral source. Thus, presenting differences associated with incurring multiple episodes only.

## Results

**Figure 1.**
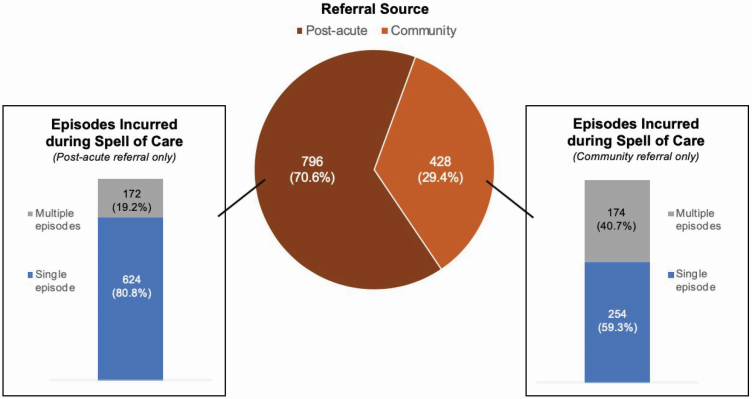
Pathways to receiving home health without a preceding hospitalization (community referral or receiving multiple episodes), among Medicare home health patients 2011–2016.

Nearly 1 in 3 (29.4%) older adults receiving Medicare home health between 2011 and 2016 were referred from the community and 1 in 4 (25.5%) incurred multiple home health episodes during a sustained spell of care (Figure 1). Nearly half (40.7%) of community-referred patients incurred multiple home health episodes during a sustained spell of care as compared with 1 in 5 (19.2%) postacute patients.

Patient characteristics *before* home health varied significantly by referral source, regardless of whether the patient incurred multiple episodes. Patients referred from the community were more socially vulnerable, had poorer health status, and received greater levels of family caregiver assistance before home health (Table 1). Among patients receiving a single episode of home health care, those referred from the community were twice as likely to be Medicaid-enrolled (24.0% vs 12.5%, *p* < .001), to have dementia (29.5% vs 12.4%, *p* < .001), to receive 80 or more hours/month of family caregiver assistance (20.7% vs 10.1%, *p* < .001), and to receive family caregiver assistance with self-care tasks (35.0% vs 19.1%, *p* < .001) or medication management (24.0% vs 12.3%, *p* < .001). Similar but slightly attenuated differences in characteristics by referral source were observed among patients who incurred multiple episodes. For example, among patients receiving multiple episodes of home health care, those referred from the community were more likely to be Medicaid-enrolled (27.2% vs 16.9%, *p* = .001), to have dementia (31.1% vs 18.4%, *p* < .01), and to receive 80 and more hours/month of family caregiver assistance (24.0% vs 14.2%, *p* = .02).

**Table 1.** Characteristics of Medicare Home Health Patients With Postacute Versus Community Referral, Stratified by Number of Episodes During Spell of Care (unweighted *n* = 1,224; weighted *n* = 5,913,080)

	Single Episode			Multiple Episodes		
Older Adult Characteristics, *n* (%)	Postacute Referral (*n* = 624)	Community Referral (*n* = 254)	*p* Value*	Postacute Referral (*n* = 172)	Community Referral (*n* = 174)	*p* Value*
**Characteristics before home health**						
*Sociodemographic characteristics*						
Age in years^†^	79.1 (0.33)	81.9 (0.65)	<.001	78.9 (0.61)	80.6 (0.63)	.04
Male sex	249 (41.4)	91 (39.5)	.69	67 (39.6)	64 (40.2)	.92
White race	469 (86.5)	146 (69.5)	<.001	107 (75.9)	99 (70.8)	.32
Medicaid-enrolled	88 (12.5)	67 (24.0)	<.001	43 (16.9)	53 (27.2)	.001
*Health status*						
Self-reported health						
Excellent/very good	175 (31.5)	48 (18.5)	<.01	32 (20.6)	30 (18.6)	.38
Good	197 (30.9)	85 (32.7)		54 (35.3)	47 (29.7)	
Fair/poor	252 (37.6)	121 (48.8)		86 (44.0)	97 (51.7)	
Number of chronic conditions^†,‡^	0.62 (0.05)	0.75 (0.08)	.13	0.81 (0.09)	0.90 (0.12)	.52
Probable dementia	102 (12.4)	81 (29.5)	<.001	40 (18.4)	63 (31.1)	<.01
Hospitalized in past year	188 (27.0)	74 (30.8)	.32	67 (36.7)	52 (31.1)	.39
*Caregiver assistance*						
Number of family caregivers^†^	1.49 (0.06)	1.87 (0.11)	<.001	1.63 (0.11)	2.05 (0.10)	.01
Receives 80+ hours/mo of family caregiver assistance	79 (10.1)	57 (20.7)	<.001	35 (14.2)	45 (24.0)	.02
Received family caregiver help with:						
Household chores	312 (46.6)	172 (62.7)	<.001	103 (54.8)	118 (66.2)	.02
Mobility	152 (19.1)	101 (34.2)	<.001	63 (28.5)	73 (39.9)	.02
Self-care tasks	142 (19.1)	98 (35.0)	<.001	58 (26.0)	73 (40.3)	<.01
Medication management	95 (12.3)	72 (24.0)	<.001	41 (17.9)	53 (25.5)	.10
**Characteristics during home health**						
Clinical severity						
Low	175 (27.9)	86 (32.7)	.44	30 (16.6)	42 (21.7)	.43
Moderate	264 (44.0)	95 (38.9)		66 (35.1)	63 (36.4)	
High	185 (28.1)	73 (28.4)		76 (48.3)	69 (41.9)	
Functional impairment						
Little or no	95 (16.3)	48 (22.4)	.19	17 (13.4)	32 (18.5)	.22
Moderate	411 (65.6)	154 (61.4)		100 (54.8)	102 (58.5)	
High	118 (18.1)	52 (16.2)		55 (31.8)	40 (23.0)	
Cognitive impairment						
Little or no	360 (64.0)	112 (47.5)	<.001	83 (49.6)	63 (39.0)	.17
Moderate	200 (28.6)	98 (36.7)		65 (34.5)	80 (45.3)	
High	64 (7.4)	44 (15.9)		24 (15.9)	31 (15.8)	

*Notes:* Data drawn from linked National Health and Aging Trends Study, Outcomes and Assessment Information Set, and Medicare claims 2011–2016. Percentages were weighted to account for survey design.

*p values are the result of Rao–Scott chi-square tests (for categorical variables) and weighted *t* tests (for continuous variables) of differences between groups.

^†^Mean (*SE*).

^‡^Chronic conditions included heart attack in the previous year, heart disease, high blood pressure, diabetes, lung disease, and stroke in the previous year.

Patient characteristics *during* home health varied significantly by number of episodes received (single vs multiple), regardless of referral source (Table 2). Among patients with a postacute referral to home health, those who incurred multiple episodes of care were more likely to have high clinical severity (48.3% vs 28.1%, *p* < .001), high functional impairment (31.8% vs 18.1%, *p* < .01), and high cognitive impairment (15.9% vs 7.4%, *p* < .001) during home health, compared to those who incurred a single episode of care. Similarly, among patients with a community referral, those who incurred multiple episodes were more likely to have high clinical severity (41.9% vs 28.4%, *p* = .01) during home health, when compared with those who incurred a single episode of care.

**Table 2.** Characteristics of Medicare Home Health Patients Incurring Multiple Versus Single Episode During Spell of Care, Stratified by Referral Source (unweighted *n* = 1,224; weighted *n* = 5,913,080)

	Postacute Referral			Community Referral		
Older Adult Characteristics, *n* (%)	Single Episode (*n* = 624)	Multiple Episodes (*n* = 172)	*p* Value*	Single Episode (*n* = 254)	Multiple Episodes (*n* = 174)	*p* Value*
**Characteristics before home health**						
*Sociodemographic characteristics*						
Age in years^†^	79.1 (0.33)	78.9 (0.61)	.82	81.9 (.65)	80.6 (.63)	.07
Male sex	249 (41.4)	67 (39.6)	.70	91 (39.5)	64 (40.2)	.92
White race	469 (86.5)	107 (75.9)	<.01	146 (69.5)	99 (70.8)	.82
Medicaid-enrolled	88 (12.5)	43 (16.9)	.13	67 (24.0)	53 (27.2)	.48
*Health status*						
Self-reported health						
Excellent/very good	175 (31.5)	32 (20.6)	.05	48 (18.5)	30 (18.6)	.80
Good	197 (30.9)	54 (35.3)		85 (32.7)	47 (29.7)	
Fair/poor	252 (37.6)	86 (44.0)		121 (48.8)	97 (51.7)	
Number of chronic conditions^†,‡^	0.62 (0.05)	0.81 (0.09)	.07	0.75 (0.08)	0.90 (0.12)	.23
Probable dementia	102 (12.4)	40 (18.4)	.07	81 (29.5)	63 (31.1)	.77
Hospitalized in past year	188 (27.0)	67 (36.7)	.04	74 (30.8)	52 (31.1)	.97
*Caregiver availability/assistance*						
Number of family caregivers^†^	1.49 (0.06)	1.63 (0.11)	.23	1.87 (0.11)	2.05 (0.10)	.24
Receives 80+ hours/mo of family caregiver assistance	79 (10.1)	35 (14.2)	.10	57 (20.7)	45 (24.0)	.46
Received family caregiver help with:						
Household chores	312 (46.6)	103 (54.8)	.10	172 (62.7)	118 (66.2)	.52
Mobility	152 (19.1)	63 (28.5)	<.01	101 (34.2)	73 (39.9)	.29
Self-care tasks	142 (19.1)	58 (26.0)	.08	98 (35.0)	73 (40.3)	.22
Medication management	95 (12.3)	41 (17.9)	.07	72 (24.0)	53 (25.5)	.78
**Characteristics during home health**						
Clinical severity						
Low	175 (27.9)	30 (16.6)	<.001	86 (32.7)	42 (21.7)	.01
Moderate	264 (44.0)	66 (35.1)		95 (38.9)	63 (36.4)	
High	185 (28.1)	76 (48.3)		73 (28.4)	69 (41.9)	
Functional impairment						
Little or no	95 (16.3)	17 (13.4)	<.01	48 (22.4)	32 (18.5)	.25
Moderate	411 (65.6)	100 (54.8)		154 (61.4)	102 (58.5)	
High	118 (18.1)	55 (31.8)		52 (16.2)	40 (23.0)	
Cognitive impairment						
Little or no	360 (64.0)	83 (49.6)	<.01	112 (47.5)	63 (39.0)	.20
Moderate	200 (28.6)	65 (34.5)		98 (36.7)	80 (45.3)	
High	64 (7.4)	24 (15.9)		44 (15.9)	31 (15.8)	

*Notes:* Data drawn from linked National Health and Aging Trends Study, Outcomes and Assessment Information Set, and Medicare claims 2011–2016. Percentages were weighted to account for survey design.

*p values are the result of Rao–Scott chi-square tests (for categorical variables) and weighted *t* tests (for continuous variables) of differences between groups.

^†^Mean (*SE*).

^‡^Chronic conditions included heart attack in the previous year, heart disease, high blood pressure, diabetes, lung disease, and stroke in the previous year.

## Discussion

We find striking differences in Medicare home health patient characteristics based on whether they accessed home health without an immediately preceding hospitalization, as well as the pathway by which they entered home health without a preceding hospitalization. Relative to postacute patients, those referred from the community were more likely to be dually enrolled in Medicaid, living with dementia, and to have relied more heavily on family caregiver assistance before home health. Relative to patients receiving a single episode, those incurring multiple episodes had greater clinical severity. Results suggest that recent changes in Medicare reimbursement for home health that reduce payment for episodes without a preceding hospitalization may threaten access to care for older adults with greater social vulnerability, ongoing functional needs, and/or clinical severity.

Prior Medicare home health payment system revisions have had the unintended consequence of disproportionately reducing access for beneficiaries with greater functional impairment, frailty, and social vulnerability (9,11–13). The substantial challenges to accessing home-based care through private payment (21,22) or Medicaid (23–26) raise concerns about the potential implications of recent Medicare home health payment changes. By reducing payments for home health episodes that are not being used to meet a short-term, postacute care need, these payment changes may threaten older adults’ ability to meet their care needs in the community setting. This possibility is especially concerning given that unmet care needs among community-dwelling older adults have been linked to increased Medicare spending (27) and risk of hospitalization and institutionalization (28–30). Additionally, our finding that community-admitted patients are disproportionately reliant on support from family and unpaid caregivers suggests that reducing reimbursement for community admissions to home health care may have important consequences for both patients and their family caregivers. The substantial demands on family caregivers providing community-based support (31–33), and the associated physical, financial, and emotional costs due to intensive caregiving (32,34–36), are of great concern. Evaluations of these recent home health payment changes should investigate how reduced reimbursement for community-referred episodes may affect or burden existing caregiving networks.

A 2019 report by Wysocki and Cheh determined that, when comparing home health patients with versus without a preceding hospitalization on factors including therapy visits received, clinical severity, and functional impairment, observed differences were largely driven by variation between those incurring single versus multiple episodes, rather than the variation between those with a postacute versus community referral. However, the authors did not examine characteristics measured before home health (6). When comparing patients by referral source (postacute vs community), we also found few significant differences in patient characteristics *during* home health, but observed a number of meaningful differences in sociodemographic factors, health status, and receipt of caregiver assistance *before* home health. Our findings suggest that contextual characteristics before home health may have a greater bearing on how patients are initially referred to home health care, whereas characteristics during home health may be more closely associated with the number of episodes received. This indicates that, while preceding hospitalization may be a valuable indicator of a home health patient’s likely care needs and resource utilization, it is far from the only important factor; patterns of home health utilization and the characteristics that drive them are far more complex.

The lack of association between patient characteristics before home health and whether the patient incurs a single episode or multiple episodes is particularly notable in light of evidence that the upward trend in home health without a preceding hospitalization is due in large part to a greater proportion of home health patients receiving multiple episodes, rather than an influx of community referrals (6). While there is no limit on the number of episodes a home health patient may receive, some have raised concerns that the shift toward multiple episodes indicates the use of Medicare-funded home health as a substitute for other sources of community-based long-term care (1,6). Our study points to greater clinical severity, regardless of referral source, and greater functional and cognitive impairment, among those with a postacute referral, as the characteristics associated with incurring multiple episodes. Although additional investigation is warranted, our results do not support the view that the growing proportion of home health episodes without an immediately preceding hospitalization indicates inappropriate use of home health solely to meet long-term care needs.

There is a lack of consensus among policymakers, researchers, and home health industry experts regarding the appropriate role for home health in meeting the needs of Medicare beneficiaries (1,37,38). Some view home health as an important source of support for older adults aging in place with ongoing care needs stemming from multiple chronic conditions and functional limitations (37,38). Others envision home health as a benefit more closely tied to temporary needs for skilled care and have raised concerns about the potential substitution of home health care for long-term care (1,37,38). Efforts to clearly define the role of home health are complicated by the myriad of care needs met by the home health benefit and a patient population with significant social and clinical complexity (39). Our findings indicate that Medicare beneficiaries access home health to meet a diverse set of care needs and support the importance of examining contextual factors when characterizing the complex patterns of home-based care utilization among high-need older adults.

### Limitations

This descriptive analysis sought to contribute new knowledge regarding the types of Medicare beneficiaries most likely to be affected by recent home health payment changes. This work is subject to several limitations that merit comment. First, as reductions in payment for home health episodes not immediately preceded by a hospitalization do not apply to Medicare Advantage coverage, we limit our sample to Medicare fee-for-service enrollees and findings may not be applicable to the Medicare Advantage population. Second, comprehensive information about individual and contextual factors preceding the home health episode was drawn from NHATS, resulting in a relatively small analytic sample. However, with sample weights, estimates are nationally representative. Finally, we consider index spells of home health care within our observation period. Therefore, our findings may not be applicable to later spells of care among beneficiaries who incur multiple spells of home health care across a given year or years.

## Conclusions

There is meaningful variation in the characteristics of older adults accessing Medicare home health by both referral source and number of episodes received. While recent Medicare home health payment system revisions reduce reimbursement for all except postacute, single episode instances of home health use (2), those referred to home health from the community or who receive multiple home health episodes also have significant care needs. We find that contextual factors before home health are more closely associated with home health patients’ referral source and that characteristics during home health are more closely linked to the number of episodes received. Ongoing decision-making surrounding reimbursement and regulation of Medicare home health care should consider the diversity of the patient population served by this benefit, and CMS should monitor whether recent reimbursement changes reduce access to care for select groups of high-need beneficiaries.
